# Exploring the Link Between Pancreatic Neuroendocrine Tumors and Depression: A Case Study

**DOI:** 10.7759/cureus.44363

**Published:** 2023-08-30

**Authors:** Nicole Ann E Villa, Gina Maria P Fiore, Eduardo D Espiridion

**Affiliations:** 1 Psychiatry, Drexel University College of Medicine, Wyomissing, USA; 2 Psychiatry, Reading Hospital, Tower Health Systems, West Reading, USA; 3 Psychiatry, West Virginia School of Osteopathic Medicine, Lewisburg, USA; 4 Psychiatry, Drexel University College of Medicine, Philadelphia, USA; 5 Psychiatry, Philadelphia College of Osteopathic Medicine, Philadelphia, USA

**Keywords:** detection, temporal relationship, neuroendocrine tumor, pancreatic neuroendocrine cancer, pancreatic cancer, depression

## Abstract

This case probes the potential temporal relationship between pancreatic neuroendocrine tumor (PNET) and depression. This patient has chronic symptoms of depression with no formal diagnosis until within a year of doctors suspecting her diagnosis of pancreatic cancer. An excisional biopsy confirmed a grade 1 neuroendocrine tumor (NET) in the pancreas, and postoperative psychiatric consultation confirmed continued elevated depression. This report presents an illustrative example of the ongoing research questions surrounding the relationship between the timing of a depression diagnosis and a PNET diagnosis. The depression-before-diagnosis relationship in pancreatic cancer patients is an observation that warrants further studies as depression could be a valuable early warning sign of pancreatic cancer.

## Introduction

Pancreatic cancer has one of the lowest five-year survival rates [1] and is the third leading cause of cancer death [2] in the United States, where this high mortality rate is attributed to poor early detection. Often, pancreatic cancer is not detected until it is in an advanced stage as low-grade tumors do not cause distinctive symptoms, if they present any at all [3].

Neuroendocrine tumors (NETs), a relatively rare type of cancer, can be found in the pancreas. The incidence rate of pancreatic neuroendocrine tumors (PNETs) is around two per one million individuals [4], and these types of tumors can be classified as either 1) functioning or hormone-secreting or 2) non-functioning or not hormone-secreting [4,5]. Between these classifications, the vast majority of NETs are non-functional [4]. A link between functioning NETs and depression has been previously explored [5-8]; however, there are limited reports on the association between non-functioning NETs and depression [9].

Generally, a temporal link between a depression diagnosis and any type of cancer diagnosis has been found [10], where this relationship was found to be most prevalent among pancreatic cancer patients [11]. This relationship, while long studied, is still not well understood. In addition, only a few studies specifically examine the link between PNETs and the onset of depression. Thus, this report presents a case of concurrent depression and pancreatic neuroendocrine cancer and probes the potential temporal relationship between PNET and worsening depression given the timing of both diagnoses.

## Case presentation

The patient is a 58-year-old divorced female who was ordered to have a psychiatric evaluation for worsening depression and anxiety symptoms. She went through an excisional biopsy of a tumor in her pancreas. The treatment team providers were considering a diagnosis of pancreatic neuroendocrine cancer.

She has a prior psychiatric history but has no prior psychiatric hospitalizations. The patient acknowledged chronic depression and anxiety symptoms, which she attributed primarily to her living situation and poor social support system. She started to experience depressive symptoms at 18 but did not experience any significant functional impairment; she did not seek psychiatric help. She lives in a trailer with a live-in partner and previously worked as a home health caregiver. She has a family history of alcohol abuse. The patient denied any personal history of alcohol or substance use disorders. In addition, she has a history of seizures, gastroesophageal reflux disease (GERD), and sleep apnea. Patient medications included sodium docusate, ondansetron, and pantoprazole. She has a history of carbamazepine treatment for a benign childhood seizure. She could not recall when that medicine was discontinued. The patient noted symptoms of sad mood and lack of interest in crocheting, an activity she typically loves to engage in.

**Figure 1:**
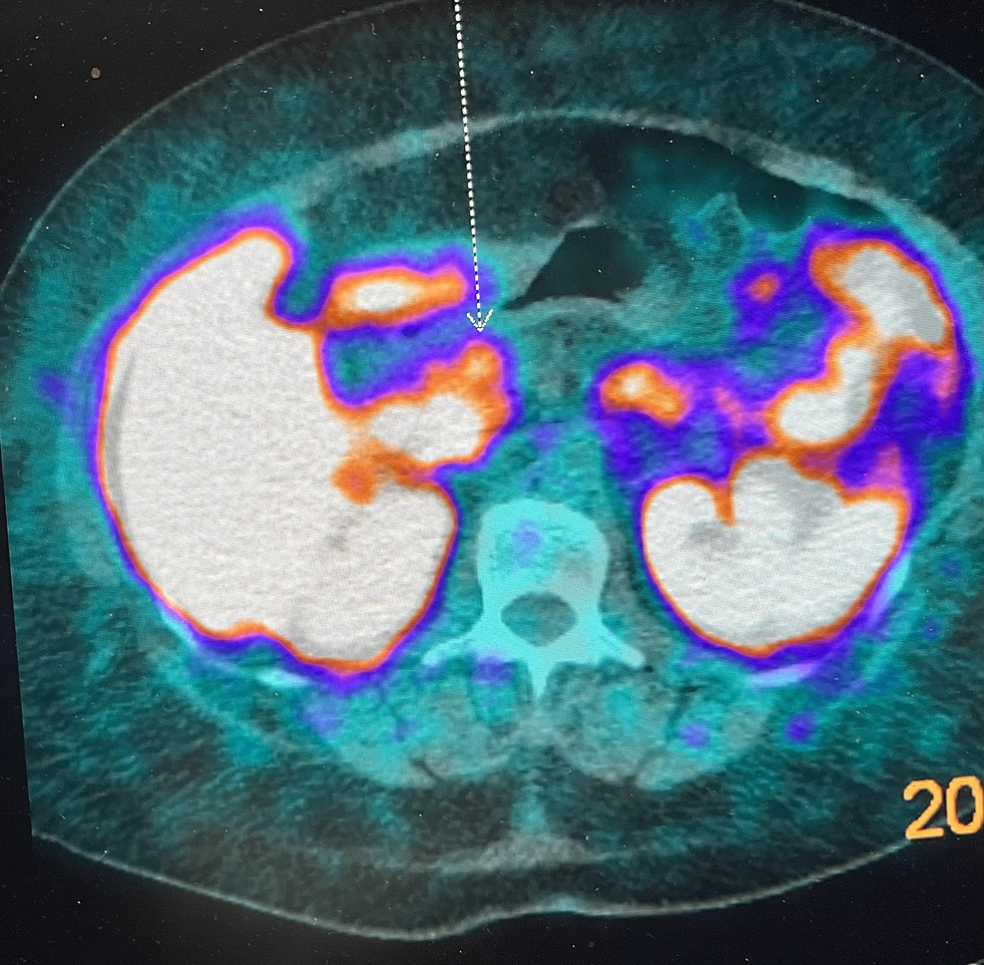
PET scan demonstrates activity in the 9 mm pancreatic uncinate process mass PET: positron emission tomography

Eight months ago, she was seen by a surgeon, and medical workups were ordered. Positron emission tomography (PET) scans showed a small, solid, hyper-enhancing 9×9 mm nodule in the uncinate process of the pancreas (Figure 1). A small neuroendocrine islet cell tumor was suspected. This finding made her very anxious and depressed, and she started to experience ruminative worries and difficulties going to sleep. She then noted social withdrawal and inappropriate guilt. She consulted her primary care physician approximately two months later, and she was started on sertraline. She reported persistent depression with sad mood, anhedonia, insomnia, poor appetite, low energy level, poor concentration, and psychomotor agitation. The patient also reports mood lability and ruminative worries. She started to experience crying spells with no known triggers. She remained alert and oriented times three with no problems in her memory and concentration. The patient has been compliant with sertraline medication, and the dose was titrated up to 100 mg per day. She never had an antidepressant trial prior to sertraline. Despite medication and treatment compliance, her symptoms were getting progressively worse, but there were no suicidal or homicidal ideations reported. In addition to the cancer diagnosis, she is stressed by poor social support and the associated pain symptoms. She notes that there is a history of physical abuse by her ex-husband. She was kept on her current sertraline dose, and a referral was made to a community mental health center for individual and couple psychotherapy, as well as case management for her complex medical and psychiatric problems.

She underwent an exploratory laparotomy and excisional biopsy of the tumor. Surgical pathology report showed a positive immunohistochemical stain for synaptophysin, and the immunohistochemical stain for Ki67 showed a proliferation index of less than 2%, which is consistent with a grade 1 well-differentiated neuroendocrine cancer. This case was discussed in an intradepartmental consensus conference with an agreement.

## Discussion

As early as the 1930s, a possible connection between pancreatic cancer and depression, where depression may be a preliminary symptom or warning sign of the cancer rather than the result of the new physical and emotional burden from the cancer, has been explored [3,12]. Further studies from the 1960s up to the present day have shown that depression often precedes a diagnosis of pancreatic cancer and that pancreatic cancer is linked with higher rates of depression than other types of cancers [11,13-16]. These considerations are particularly interesting in the case of this patient. While the patient did report chronic symptoms of depression and anxiety, her depression appeared to worsen in the months prior to her cancer diagnosis. Her depression became increasingly severe and showed no improvement despite being given psychiatric medication leading up to the excisional biopsy, which confirmed the suspected diagnosis of a pancreatic neuroendocrine tumor (PNET). The timing of the exacerbation of depression prior to the pancreatic cancer diagnosis and the body of literature around this connection raise questions on the temporal relationship between depression and PNET.

The depression-before-diagnosis relationship in pancreatic cancer is not well understood; however, some studies have formed different hypotheses: Depression may be a signal of a developing tumor, the depression predisposes patients to develop cancer due to straining the immune system, or there is some interplay of these two theories [17]. Recent studies have looked at the timing of the cancer diagnosis compared to the onset of depression and found that patients with pancreatic cancer had higher rates of depression in the year leading up to their cancer diagnosis compared to cancer-free controls [16]. A study in the United States involving over 62,000 patients found similar results, where more than half of the subjects in the study had comorbid depression with pancreatic cancer and the vast majority were diagnosed with depression within six months either before or after the cancer diagnosis [11]. Although there is evidence showing increased psychiatric diagnosis around the time of diagnosis of any form of cancer [10], a more recent study by Seoud et al. found that pancreatic cancer had higher rates of depression diagnosis within six months of cancer diagnosis compared to other cancers such as breast, prostate, lung, and colorectal [11]. The patient’s exacerbated depression coinciding with her cancer diagnosis fits in well with the most recent literature exploring the depression-before-diagnosis relationship in pancreatic cancer. However, there are other factors that need to be considered when examining what caused her increased depression symptoms.

Given the pancreatic location of the cancer and its classification as a neuroendocrine tumor, these factors should be considered when evaluating possible contributing factors to the patient’s increased depression. A correlation between depression, anxiety, and gastroenteropancreatic NETs (GEP NETs) has been demonstrated in other papers, with rates of depression near 20% among GEP NET patients studied [9]. One study suggests that PNET patients, in particular, have higher rates of depression with mild to moderate symptoms found in about 40% of PNET patients surveyed [18]. Although the literature on this specific relationship is sparse, these findings linking GEP NETs to increased rates of depression make the patient’s neuroendocrine type of cancer a significant consideration for the cause of her increased depressive symptoms. Further research connecting depression with NETs in primary sites not including the pancreas would help clarify the impact that the neuroendocrine nature of the tumor has on depression and separate that from the contributing factor of location in the pancreas.

Additionally, with NETs, there is a chance that the hormone imbalance from tumor secretions can lead to secondary effects such as depression. This phenomenon has been especially studied in patients with carcinoid syndrome due to patients having a functioning NET that secretes serotonin [5-7]. However, in the case of this patient, as is the case with 90% of PNET patients, the tumor appears to be non-functioning or does not secrete any hormones [4].

Among non-functioning PNETs and non-endocrine tumors in the pancreas, biological explanations for the link between depression and the cancer include various inflammatory, hormonal, immunological, and biochemical markers; however, no definitive explanation has been identified [3]. A significant relationship between depression and interleukin 6 (IL-6) cytokines and between IL-6 cytokines and pancreatic cancer has been found, but there was no data to support that these cytokines played a role in causing the depression in the pancreatic cancer patients [19]. Given the poor understanding of the biological relationship between pancreatic cancer and depression and the other significant known stressors in our patient’s life, it is important not to discount the multifactorial causes of her depression. Her lack of social support and long-standing undesirable living situation have likely decreased her resilience to the additional psychological distress of a cancer diagnosis. Decreased resilience, where resilience is defined as an individual’s ability to maintain or restore relatively stable psychological and physical functioning when confronted with stressful life events, is associated with poorer mental health outcomes in cancer patients [20].

The preexisting stressors in this patient’s life and the significant burden of receiving a cancer diagnosis are all reasonable triggers for the exacerbation of her depression. However, because the coinciding timing of the two diseases aligns with the larger, continuing investigation into the relationship between pancreatic cancer and depression, it raises questions as to whether a temporal relationship between the two diseases is also observed here with this patient. The biggest indicator that this case may be representative of the larger, ongoing pattern seen in recent studies where a depression diagnosis precedes pancreatic cancer is that the patient’s depressive symptoms heightened before she received any formal diagnosis of her cancer. In the big picture, given the high mortality rate and poor early detection of pancreatic cancer, the possible temporal relationship between pancreatic cancer and depression is interesting in its potential to serve as an early warning sign of pancreatic cancer, and further studies should continue to investigate this. At the very least, further research into this depression-before-diagnosis relationship could improve our understanding of one of the deadliest cancers and hopefully lead to improved outcomes for future patients. It would also be beneficial to do routine depression screening on all patients with malignancies.

## Conclusions

In the broader context of all pancreatic cancers, this case report presents an illustrative example of the ongoing research and questions surrounding the relationship between the timing of a depression diagnosis and a pancreatic cancer diagnosis. Given the importance of early diagnosis in pancreatic cancer patients, further studies should be done to determine if depression could be a valuable early warning sign.
